# Supplementary material for: Detection of Chikungunya virus in bodily fluids: The INOVACHIK cohort study
Source: PLoS Negl Trop Dis. 2022 Mar 7;16(3):e0010242. doi: 10.1371/journal.pntd.0010242 (PMC8929700; doi:10.1371/journal.pntd.0010242)
Supplement: Attachment — Submitted filename: Response to Reviewers_04.01.2022.docx [file pntd.0010242.s001.docx]

Manuscript number: **PNTD-D-21-01132**

Title: **Detection of Chikungunya virus in bodily fluids: The INOVACHIK Cohort Study.**

**PLOS NEGLECTED TROPICAL DISEASES**

**Corresponding author:** Ezequias Batista Martins

| REVIEWER COMMENTS | RESPONSE |
| --- | --- |
| **REVIEWER 1** | |
| 1. The presentation of the data in Table 2 is confusing. Is this data derived from the initial sample collection at the time of enrollment? Or is it combined data from multiple collections? In some cases, the text says “in at least one specimen” or other similar statements. The presentation of these data needs to be clarified and improved. In addition, it also seems important to include information about the time of disease onset relative to collection of the initial samples. | Thank you for your remarks with which we completely agree. We have made new formatting for table 2 (now Table 3), allowing a better understanding of the data.  Data are derived from the combination of all study visits. The first samples (for all fluids) were collected within the first week after the onset of symptoms. After that, samples were collected every 15 days for 2 months and at 3 months of follow-up. Each participant collected a maximum of 6 samples for each body fluid in the study.  We have added more information about the time of disease onset according to the collection of the initial samples:  “Patients with symptoms reported for up to 7 days were included in the study. The first visit (with fluid collection) was performed on different days, depending on the patient's arrival at our clinic.”  “The first samples for all fluids were collected in the first week of symptoms, and the second samples were collected within 14 days after the onset of symptoms (+/- 3 days as visit window). Data regarding clinical signs and symptoms was collected during the acute phase (1–14 days)." |
| 2. Which collection times were used for the persistence analysis and which ones were used for the acute analysis (is this Table 2?). This should be clarified. | The first samples for all fluids were collected in the first week of symptoms, and the second samples were collected within 14 days after the onset of symptoms (+/- 3 days as visit window). Data regarding clinical signs and symptoms was collected during the acute phase (1–14 days). All patients were tested over the study duration (3 months), despite undetectable RT-PCR results in all body fluids collected during a visit.  We have added this information to the Methods section. |
| 3. The details of RNA isolation, cDNA generation, and the RT-PCR assay are missing and should be included in the materials and methods section. | We agree with this pertinent comment. Assay details and new references were included in the Methods section (Laboratory Tests).  “The samples were tested for CHIKV using real-time reverse transcriptase polymerase chain reaction (rRT-PCR). Following the manufacturer's instructions, RNA was extracted using the QIAmp Viral RNA Mini Kit (Qiagen, Valencia, CA, USA). The general procedures for rRT-PCR for chikungunya, zika, and dengue have been described elsewhere [21, 22, 23]. The RT-PCR mix was prepared using the GoTaq Probe 1-Step RT-qPCR System (Promega, Madison, USA) and was run using the Applied Biosystems 7500 Real-Time PCR System. Cycle threshold (Ct) values lower than 38 and sigmoid curves were considered positive.” |
| 4. The authors state that any reaction with a Ct value lower than 38 and with sigmoid curves was considered positive. It would improve the study to include the Ct values detected to give readers an opportunity to understand the strength of the signal in the different body fluids over time. | Thank you for your remarks with which we completely agree. We have added Figure 3, reporting CHIKV Cycle Threshold (Ct) by days after the onset of symptoms in Serum, Urine, Saliva, and Vaginal Secretion. |
| 5. Given the sensitivity of RT-PCR and the potential for false positives, the authors should report the methods and measures used to minimize contamination and other possible sources of false positive signals. | Thank you for your pertinent suggestion. We have added two paragraphs to the Methods section (Laboratory Tests) to clarify this subject.  “As a reference laboratory, all measures to avoid cross-contamination within the samples were adopted. There were different areas designated only for PCR: 1. In two large rooms, RNA extraction (manual or automated) was performed in cabinets with UV light or in a biological safety cabinet, with an adjacent room for adding samples in the PCR mix; 2. One “clean” room was used for mix preparation only; 3. One room with thermocyclers was used for the amplifications.”  “A non-template control (NTC) was used to check for the absence of sample cross-contamination, contamination of reagents, consumables, and environment. The NTC is a negative "sample" that can be water or negative plasma extracted simultaneously with the clinical samples and included in the amplification process. In all steps, tips with barriers were used, most consumables were disposable, and gloves were changed frequently. In addition, aseptic cleaning was frequently performed in all rooms.” |
| 6. The clinical data presented from lines 166-175: Are these the signs and symptoms that were present at the time of study enrollment and first sample collection? This is not clear. | Reported signs and symptoms was referred to the enrollment and the second visit (1-14 days). We have added this information to the Methods section (Study site and cases management):  “Clinical data and biological samples were collected every 15 days for 2 months, with a final 3-month collection. The first samples for all fluids were collected in the first week of symptoms, and the second samples were collected within 14 days after the onset of symptoms (+/- 3 days as visit window). Data regarding clinical signs and symptoms was collected during the acute phase (1–14 days).” |
| 7. The authors indicate that clinical data were collected throughout the study, however, these are not reported. It would be useful to compare the clinical presentation to the decline in viral RNA signal in samples. Is there any relationship to the resolution and/or persistence of clinical symptoms and signs? | Thank you for the insightful comment. We have judged that this would be more appropriate for another manuscript (in the process) to assess factors associated with viral detection and persistence, including clinical and laboratory data. |
| **REVIEWER 2** | |
| A description of how the RNA was extracted (Trizol, RLT, AVL, etc), cDNA generation, and if any post-PCR analysis was performed is missing. | We agree with this pertinent comment. Assay details and new references were included in the Methods section (Laboratory Tests) as detailed in item 3, answers to Reviewer 1. |
| Line 111: what do the authors mean by "focus of infection"? Please clarify the specific criteria for the time post symptom onset and positive CHIKV PCR to the time of enrollment in the study? | Thank you for this comment. We have rephrased this sentence.  “Men and women aged ≥18 years who had developed acute fever or arthralgia (with or without a rash) and no evident focus of bacterial infection within the previous 7 days were enrolled”  Patients with symptoms reported for up to 7 days were included in the study. The first visit (with fluid collection) was performed on different days, depending on the patient's arrival at our clinic. We added this information to the text. |
| Line 141-142: What is the rationale or experimental data for the assumption that all the fluids would be positive at symptom onset? | There are no experimental data to confirm our assumption. However, we have assumed that if there is no other pathology involved, the initial symptoms were related to Chikungunya virus infection. Also, we have used the same criteria for a similar study on the persistence of Zika virus in body fluids published by Paz-Bailey G, et al. 2018.  Paz-Bailey G, Rosenberg ES, Doyle K, Munoz-Jordan J, Santiago GA, Klein L, et al. Persistence of Zika Virus in Body Fluids - Final Report. The New England journal of medicine. 2018;379(13):1234-43. |
| Line 144-145: What is the rationale for censoring these individuals? If they are still positive at the end of the study, this would still indicate CHIKV RNA persistence, supporting the other data that CHIKV RNA persists long-term. | Thank you for your remarks with which we completely agree. If a patient remained positive until the end of the study, he or she was counted for the survival analysis indicating the persistence of CHIKV RNA.  We have decided to remove this sentence from the Statistical analysis section: "Participants who tested positive for any fluid during the study without a negative result being yielded by the end of follow-up were censored.” |
| Table 1: Race/ethnicity data: are these the correct terminologies for different race and ethnicities? | Thank you for this comment. We have removed the word “**ethnicity**” from the table for better understanding. |
| Line 166-175: Please report the signs and symptoms in a table. | Thank you for this very appropriate suggestion. We have included a new table reporting the signs and symptoms (new Table 2). |
| Line 181-184: Please report the IgM ELISA titers in a figure. | Thank you for the insightful comment. However, we think the actual explanatory paragraph was appropriate to preset the number of reactive anti-CHIKV IgM participants. |
| Figure 2: The median and maximum time to a negative CHIKV RNA result is reported in the text, but it would be useful to have these numbers also represented in the figure or in a separate table. | Thank you for your pertinent suggestion. We have added a new table (Table 4) to show Weibull models with percentiles including medians and 95^th^ percentiles for each body fluid. |
| The introduction and conclusions/discussion could be reworked to highlight the public health significance of the work. For example, in the discussion, the authors could discuss how the urine or saliva test is less-invasive compared to blood tests for viral RNA. The downside is that the viral RNA is most consistently detected in the serum (80% positivity in this dataset). The authors could also expand upon the difference in detecting viral RNA compared to isolating infectious virus and any implications that this might have. | Thank you for this recommendation. We have added your suggestions to our discussion section. |
| Lines 26-27: Needs a little more information in the abstract background section. A statement that introduces CHIKV is missing. A statement is needed about what was done and why the detection of CHIKV in body fluids may be important for public health. | Thanks for your pertinent suggestion. The background has been modified, and more information has been added.  “**Background:** Chikungunya is a widely distributed, re-emerging tropical disease caused by the chikungunya virus (CHIKV). Little is known about the duration for which CHIK RNA are detectable in bodily fluids, especially genital secretions, and current evidence is based on small series or case reports. An understanding of viral dynamics across different body compartments can inform diagnostic testing algorithms and public health prevention interventions.” |
| Line 52: Expand on the conclusion section- what are the implications for long-term detection of CHIKV RNA in body fluids? | Thank you for this recommendation. We have added your suggestions to our discussion section. |
| Line 69: State where CHIKV is geographically distributed. | Thanks for your pertinent suggestion. The paragraph has been expanded, and information regarding geographical distribution has been added.  " Prior to 2013, CHIKV cases and some outbreaks were identified in several countries in Africa, Asia, Europe, and the Indian Ocean Islands. In 2013, the first local transmission of the CHIKV in the Americas was identified in the Caribbean countries and territories. The virus then spread throughout most of the Americas in 2014, especially in Brazil [1, 2]." |
| Line 73: CHIKV disease is generally described in three phases: acute, post-acute, and chronic. An expansion of the clinical symptoms of each phase is needed. | Thank you for pointing this out. The paragraph was modified as follows:  "Clinically, the disease has three main phases: acute, post-acute, and chronic. In the acute phase, symptom onset ranges from 2–12 days following bites by an infected mosquito. This phase is associated with an abrupt onset of fever, headache, arthralgia, myalgia, fatigue, prostration, and rash. Severe joint pain is the most prevalent symptom, described in 90% of cases. The post-acute phase appears after 14 days of illness, following the febrile period. At this stage, joint pain is observed, which may last for up to 3 months. Finally, in the chronic phase, articular manifestations are seen, which are usually debilitating and can persist for many years [5, 6]." |
| Line 83: There should be a more complete description of what has been shown for CHIKV detection from body fluids over each phase of infection. Also add the citation with Joy Gardner, et al. 2015 Plos One that showed infectious CHIKV recovery from patient saliva and compared it to animal models. | Thanks for your pertinent suggestion. Little is known about the duration of detectable Chikungunya virus (CHIKV) RNA in bodily fluids and the main references have already been cited in the introduction. The study of Gardner, *et al.* is very relevant and brings essential discoveries. We have added this reference to our introduction section.  " Gardner et al. showed that oral fluid (saliva) of CHIKV-infected animals and humans might contain infective CHIKV in the acute phase of the disease. Human saliva samples were obtained from 13 CHIKV-positive patients who presented with hemorrhagic manifestations [16]." |
| Line 83: Remove the word "other". Is anything known for other alphaviruses (RRV, ONNV, MAYV, VEEV, etc) in terms of detection in other body fluids such as saliva? | Thanks for your pertinent suggestion. The word has been removed. Studies for other alphavirus are more related to vector saliva than human saliva. |
| Line 183-184: In reference to the six participants that had a single visit with negative IgM antibodies- what was their time post symptom onset? | The lack of IgM class antibodies production in these participants was probably because sample collection was performed two days after symptom onset in five participants and three days after symptom onset in another participant. Therefore, this information was added to the Results section (Specific IgM antibodies against CHIKV). |
| Line 271-276: Expand this paragraph to discussing CHIKV transmission as a whole. Please include a statement about how transmission occurs primarily through mosquitos but we do not know if transmission may occur by saliva or sexual contact (as with ZIKV for example). | Thank you for your pertinent suggestion. More information has been added to the discussion section. |
| 1. In the introduction, there should be a more detailed discussion of what we know about CHIKV RNA detection in patient body fluids (serum, urine, saliva, etc) during each infection phase- the acute, post-acute phase, and chronic phase. In addition, CHIKV detection in body fluids from CHIKV animal models could enhance the discussion and provide some comparison. | Thank you for your pertinent suggestion. More information has been added to the introduction section. |
| 2. The long-term detection of vRNA shed in bodily fluids could reflect either infectious virus in the bodily fluids, or vRNA in the absence of infectious virus. The genome copy number would be useful because high copy numbers may be more suggestive that infectious virus is present. The authors should report CHIKV levels as genome copies (or Ct's if they don't have genome copies) relative to days after symptom onset. In addition, please describe the detection limit of the PCR assay. | Thank you for your remarks with which we completely agree. We add a new figure 3, reporting CHIKV Cycle Threshold (Ct) by days after the onset of symptoms in Serum, Urine, Saliva, and Vaginal Secretion. Also, we have added information on this subject to our discussion section. |
| 3. In Figure 2- I'm not an expert on statistics, but the Weibull curve diverges from the KM curve in the serum, urine, and saliva data, but not as much in the vaginal secretion data. Why is this the case? Are both statistical analysis needed? | The use of the Weibull curve was used as a smoother for the Kaplan-Meier estimator’s distribution. The Weibull curve showed up the better choice as a smoothing curve in common to represent the Kaplan-Meier estimator’s distribution for all studied fluids (serum, urine, and saliva data). A more detailed explanation was added to the methods section. |
| 4. The conclusions section could use more discussion of the significance of CHIKV detection in body fluids. It would also benefit from a more organized structure. For example, there could be a paragraph devoted to CHIKV RNA detection in acute phase and a separate paragraph on long-term detection. | Thank you for this recommendation. We have expanded our discussion following suggestions. |
